# A Novel Direct Factor Xa Inhibitory Peptide with Anti-Platelet Aggregation Activity from *Agkistrodon acutus* Venom Hydrolysates

**DOI:** 10.1038/srep10846

**Published:** 2015-06-02

**Authors:** Meimei Chen, Xiaohui Ye, Xin Ming, Yahui Chen, Ying Wang, Xingli Su, Wen Su, Yi Kong

**Affiliations:** 1 School of Life Science & Technology, China Pharmaceutical University, 24 Tong Jia Street, Nanjing 210009, PR China; 2 State Key Laboratory of Natural Medicines, China Pharmaceutical University, Nanjing 210009, PR China; 3 Division of Molecular Pharmaceutics, UNC Eshelman School of Pharmacy, The University of North Carolina at Chapel Hill, Chapel Hill, NC 27599, USA

## Abstract

Snake venom is a natural substance that contains numerous bioactive proteins and peptides, nearly all of which have been identified over the last several decades. In this study, we subjected snake venom to enzymatic hydrolysis to identify previously unreported bioactive peptides. The novel peptide ACH-11 with the sequence LTFPRIVFVLG was identified with both FXa inhibition and anti-platelet aggregation activities. ACH-11 inhibited the catalytic function of FXa towards its substrate S-2222 via a mixed model with a K_i_ value of 9.02 μM and inhibited platelet aggregation induced by ADP and U46619 in a dose-dependent manner. Furthermore, ACH-11 exhibited potent antithrombotic activity *in vivo*. It reduced paralysis and death in an acute pulmonary thrombosis model by 90% and attenuated thrombosis weight in an arterio-venous shunt thrombosis model by 57.91%, both at a dose of 3 mg/kg. Additionally, a tail cutting bleeding time assay revealed that ACH-11 did not prolong bleeding time in mice at a dose of 3 mg/kg. Together, our results reveal that ACH-11 is a novel antithrombotic peptide exhibiting both FXa inhibition and anti-platelet aggregation activities, with a low bleeding risk. We believe that it could be a candidate or lead compound for new antithrombotic drug development.

Thrombosis is a leading cause of morbidity and mortality throughout the world and plays a pivotal role in the pathogenesis of numerous cardiovascular disorders, including acute coronary syndrome, myocardial infarction, unstable angina, deep-vein thrombosis and pulmonary embolism^1^. Blood clotting in response to vascular injury requires the activation of zymogens in the coagulation cascade, in which thrombin and FXa are key players. FXa, a trypsin-like serine protease, functions at the convergence of the extrinsic and intrinsic coagulation pathways. Thus, it plays a central role in the coagulation cascade by catalyzing the production of thrombin, leading to further clot formation and wound closure^2^.

The search for thrombin inhibitors has been the focus of early antithrombotic drug development efforts because thrombin plays multiple roles in normal coagulation, including catalyzing the cleavage of fibrinogen to fibrin, catalyzing the formation of FVa and FVIIIa, and activating platelets^3^. In clinical trials, direct thrombin inhibitors did not block the continuous production of thrombin from prothrombin, which is the most abundant procoagulant zymogen in the coagulation cascade^4^,^5^,^6^. The high levels of thrombin inhibition necessary to produce an effective antithrombotic action *in vivo* can lead to unacceptable levels of anticoagulation and an accompanying risk of hemorrhage. In contrast, inhibition of FXa, a component of the prothrombinase complex that converts prothrombin to thrombin, would prevent the continuous production of thrombin while maintaining the basal activity of thrombin necessary for primary hemostasis^7^. Thus, FXa has emerged as a more attractive target for the development of new anticoagulants in recent years^8^. Among the numerous natural FXa inhibitors, tick anticoagulant peptide (TAP)^9^ and antistasin (ATS)^10^ have been studied in various arterial and venous thrombosis models, as well as in a model of disseminated intravascular coagulation (DIC). The results have unequivocally demonstrated that the specific inhibition of FXa is superior to that achieved by heparin or direct thrombin inhibitors^11^.

Some peptides, which are inactive within the parent protein, can be released by enzymatic hydrolysis and exhibit diverse bioactivities. Thus, a number of bioactive peptides have been obtained by enzymatic methods, and those peptides include angiotensin-converting enzyme (ACE) inhibitory peptide from tuna frame protein hydrolysate^12^, antioxidant peptide from grass carp muscle hydrolysate^13^, antimicrobial peptide from anchovy hydrolysate^14^, and anticoagulant peptide from scorpion protein and goby muscle protein hydrolysate^15^,^16^. However, studies of the hydrolysis of animal venoms are rare. Therefore, the goal of the present study was to hydrolyze snake venom, which contains numerous antithrombotic proteins and peptides, in order to release potential anti-FXa peptides. Bioassay-directed chromatographic separation was carried out in the presence of FXa inhibiting activity, and a novel peptide demonstrating both FXa inhibition and anti-platelet aggregation activities was obtained. Its antithrombotic activity was further characterized in animal models. To the best of our knowledge, this is the first report of the enzymatic hydrolysis of snake venom and the discovery of a new peptide demonstrating dual inhibition of FXa and platelet aggregation.

## Results

### Preparation of *Agkistrodon acutus* venom hydrolysates

**Figure 1:**
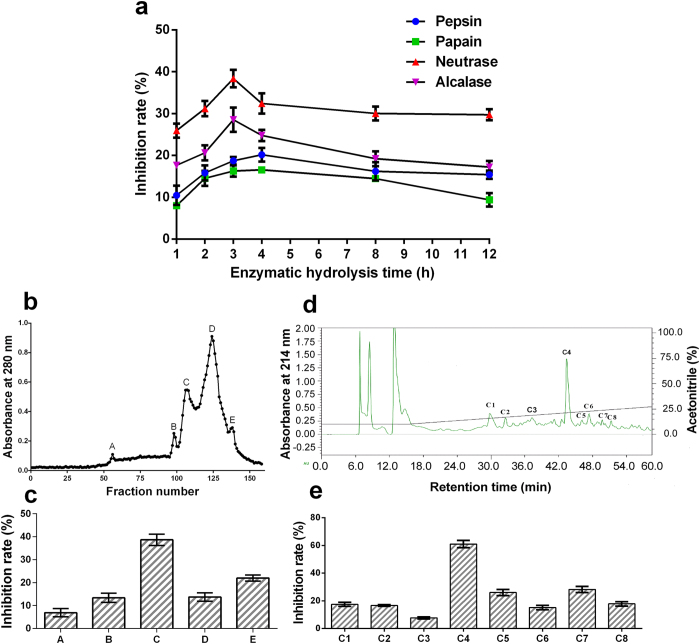
Selection of enzymatic hydrolysis conditions and bioassay-directed chromatographic separation of the FXa inhibitory peptide. (**a**) FXa inhibitory activities of *Agkistrodon acutus* venom hydrolysates obtained by treatment with pepsin, papain, neutrase and alcalase at 1, 2, 3, 4, 8, 12 h intervals. (**b**) *Agkistrodon acutus* venom hydrolysate produced by neutrase (3 h) was separated on a gel filtration column packed with Sephadex G-50 (2.6 × 100 cm). The hydrolysate was eluted by distilled water at a flow rate of 0.6 mL/min, collecting fractions of 3.6 mL. (**c**) The FXa inhibitory activities of fractions A-E. (**d**) Fraction C was separated on a Hedera ODS-2 column (20 × 250 mm). (**e**) The FXa inhibitory activities of fractions C1-C8. Data are presented as the mean ± SD (n = 3).

Enzymes have specific cleavage positions within polypeptide chains. To select suitable proteases, *Agkistrodon acutus* venom was independently hydrolyzed with pepsin, papain, neutrase and alcalase using a batch reactor. As shown in Fig. 1a, the FXa inhibitory activities of *Agkistrodon acutus* venom hydrolysates increased after digestion with various proteolytic enzymes during the first 3 or 4 hours and then decreased, which may result from excessive hydrolysis that leads to a decrease in bioactive peptide content in the hydrolysate. Amongst the four enzymes examined, neutrase digest for 3 h resulted in the highest FXa inhibitory activity, from which the maximum inhibitory rate of FXa by the hydrolysate reached 38.37 ± 0.58% (mean ± SD, n = 3) at a concentration of 5 mg/mL. Thus, the *Agkistrodon acutus* venom hydrolysate produced by neutrase treatment for 3 h was selected for further purification.

### Purification of FXa inhibitory peptide from *Agkistrodon acutus* venom hydrolysates

The purification procedure consisted of a combination of gel filtration and reverse-phase chromatography, guided by monitoring FXa inhibitory activity. The neutrase hydrolysate was dissolved in distilled water and was applied to a gel filtration column (2.6 × 100 cm) packed with Sephadex G-50 and equilibrated with distilled water. As reported in Fig. 1b, five fractions designated A-E were isolated. Each was collected and independently tested for FXa inhibitory activity. The fraction C, which displayed the strongest inhibitory effect on the amidolytic activity of FXa (Fig. 1c), was further purified by RP-HPLC on a Hedera ODS-2 column (20 × 250 mm) equilibrated with 10% acetonitrile containing 0.1% TFA. The elution profile obtained on a linear gradient of 10–30% acetonitrile containing 0.1% TFA included eight fractions designated C1-C8 (Fig. 1d). The fraction C4 displayed the highest FXa inhibitory activity (Fig. 1e) and was collected and lyophilized. The purity of fraction C4 was greater than 96%, as determined on an analytical C_18_ column (4.6 × 250 mm) using a Shimadzu LC-20AT HPLC system (Supplementary Fig. S1).

### Determination of the molecular mass and peptide sequence

**Figure 2:**
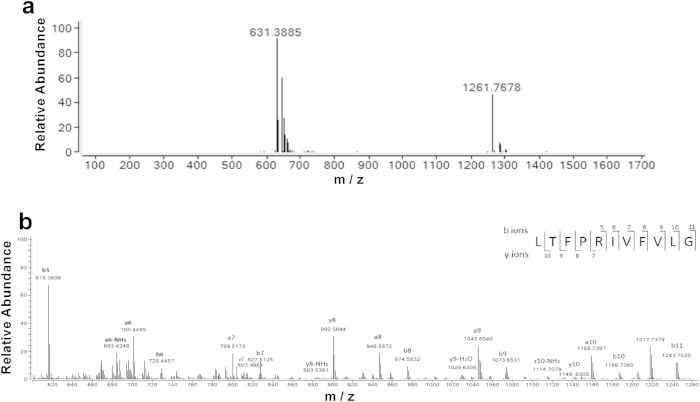
The peptide obtained from hydrolysate was identified by ESI-MS/MS mass spectrometry. (**a**) Mass spectrum of the C4 fraction. (**b**) MS/MS spectrum of ion m/z 1261.77. By manual calculation, the sequence of this peptide is displayed with the fragment ions observed in the spectrum. For clarity, only b and y ions are labeled.

The molecular mass of the purified fraction C4 was determined using ESI-MS. The m/z values of the single charged ions ([M + H]^+^) and double charged ions ([M + 2H]^2+^) were 1261.77 and 631.39 Da, respectively (Fig. 2a). Thus, the molecular mass of the peptide was 1260.77 Da. The amino acid sequence of this peptide was identified as LTFPRIVFVLG by tandem mass spectrometry (MS/MS) (Fig. 2b), which is consistent with the observed mass from ESI-MS.

In order to test its biological activity, ACH-11 was chemically synthesized using the Fmoc solid-phase synthesis method and was then purified by HPLC on a Kromasil Semipreparative C_18_ column (250 × 10 mm). The purity of ACH-11 was greater than 98% (Supplementary Fig. S2).

### ACH-11 is a novel potent FXa inhibitor

**Figure 3:**
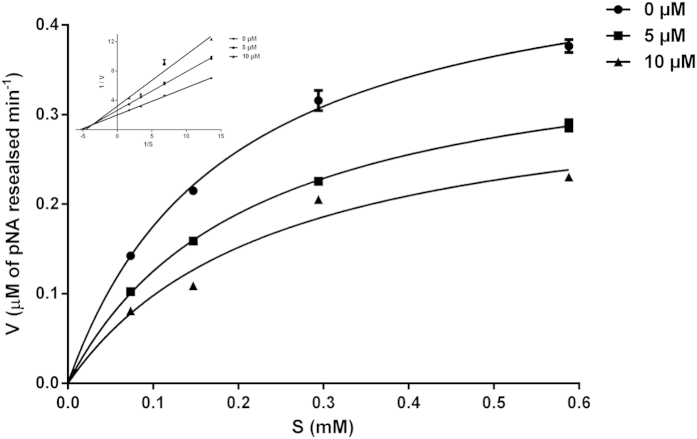
Kinetic activity of ACH-11 to FXa *in vitro*. Lineweaver-Burk plot (inset) for ACH-11 kinetics in PBS at pH 8.34 and 37 °C with mixed inhibition in the presence of different concentrations of ACH-11: 0 μM (slope of 0.376), 5 μM (slope of 0.528) and 10 μM (slope of 0.698). The plots represent the mean of 3 independent measurements.

ACH-11 inhibited the catalytic activity of FXa towards its substrate S-2222 in a dose-dependent manner, and the Michaelis-Menten plot is shown in Fig. 3. Plots of its inhibition activity were obtained for different concentrations of ACH-11 and showed a slope of 0.376 for the control without ACH-11, a slope of 0.528 for 5 μM, and a slope of 0.698 for 10 μM.

The Lineweaver**-**Burk plots in Fig. 3 (inset) showing inhibition of FXa by ACH-11 indicated a mixed type of inhibition. A decrease in the V_max_ value with a subsequent increase in the K_m_ value of FXa towards S-2222 in the presence of ACH-11 was observed. The K_i_ value for FXa inhibition towards S-2222 by ACH-11 was determined to be 9.02 μM.

### ACH-11 exerted anticoagulation activity both *ex vivo* and *in vivo*

**Figure 4:**
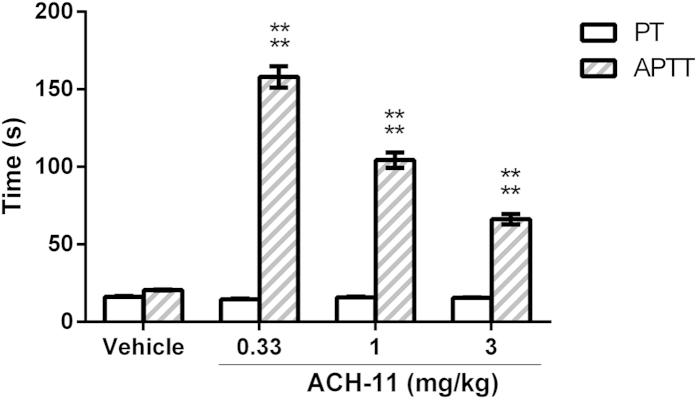
ACH-11 prolonged *ex vivo* plasma coagulation time. Thirty minutes after the administration of ACH-11 (3, 1 and 0.33 mg/kg, i.v.) and the vehicle, blood was taken from the inferior vena cava of rats, and platelet-poor plasma (PPP) was then obtained by centrifugation at 2,000 g for 10 min at room temperature to test *ex vivo* PT and aPTT. Data are presented as the mean ± SD of three independent experiments. ^****^P < 0.0001 *versus* vehicle, analyzed by one-way ANOVA, followed by the Tukey multiple comparison test.

To evaluate the effects of ACH-11 on coagulation parameters *ex vivo*, we measured the influence of the peptide on the activated partial thromboplastin time (aPTT) and prothrombin time (PT). Intravenous pre-administration of ACH-11 to rats significantly increased aPTT in a dose-dependent manner at doses ranging from 0.33 to 3 mg/kg. As shown in Fig. 4, aPTT and PT in the vehicle-treated group were 20.58 ± 0.33 s and 16.18 ± 0.62 s (mean ± SD, n = 3), respectively. In the ACH-11-treated groups, aPTT increased significantly to 158.05 ± 6.89 s, 104.35 ± 4.98 s and 62.20 ± 3.32 s at doses of 3, 1 and 0.33 mg/kg (P < 0.0001 *versus* vehicle), respectively. Even at the highest test dose of 3 mg/kg, PT did not significantly increase when compared to the vehicle group (P > 0.05).

In *in vivo* clotting time experiments, ACH-11 significantly prolonged blood clotting time in a dose-dependent manner (P < 0.0001 *versus* vehicle, n = 10) at doses ranging from 0.33 to 3 mg/kg. This indicated that ACH-11 had anticoagulant effects *in vivo* (Supplementary Fig. S3).

### ACH-11 inhibited ADP- and U46619-induced rabbit platelet aggregation *in vitro*

**Figure 5:**
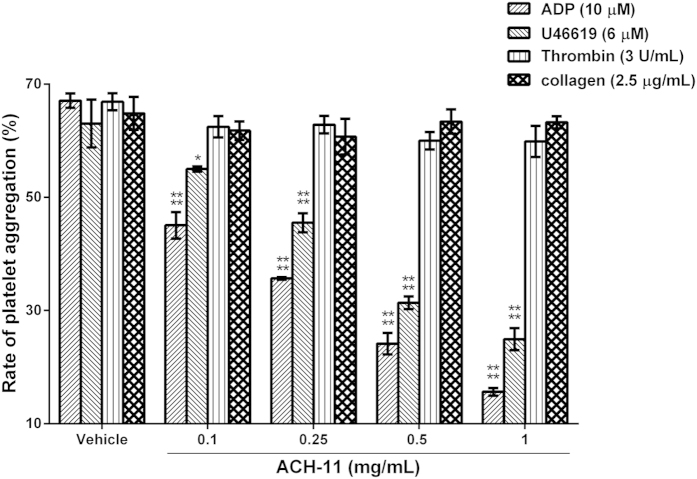
ACH-11 inhibited ADP- and U46619-induced rabbit platelet aggregation *in vitro*. PRP was preincubated for 5 min with different concentrations of ACH-11 (0.1, 0.25, 0.5 and 1 mg/mL) or the vehicle. Platelet aggregation was initiated with ADP (10 μM), U46619 (6 μM), thrombin (3 U/mL) or collagen (2.5 μg/mL). Data are the mean ± SD (n = 3), ^****^P < 0.0001 and ^*^P < 0.05 *versus* vehicle, analyzed by one-way ANOVA, followed by the Tukey multiple comparison test.

To study the effect of ACH-11 on platelet aggregation, we used rabbit platelet-rich plasma (PRP) induced with various agonists as an *in vitro* model. The results showed that ACH-11 selectively inhibited platelet aggregation induced by different agonists. As shown in Fig. 5, ACH-11 inhibited ADP (10 μM)- and U46619 (6 μM)-induced platelet aggregation in a dose-dependent manner, with IC_50_ values of 204.24 μM (95% CI, 185.36–225.02 μM) and 467.57 μM (95% CI, 415.62–525.95 μM), respectively. At 1 mg/mL, the peptide inhibited ADP (10 μM)- and U46619 (6 μM)-induced rabbit platelet aggregation by 76.69 ± 0.99% and 60.46 ± 3.08% (mean ± SD, n = 3), respectively, while ACH-11 exerted only weak inhibitory effects on thrombin (3 U/mL)- and collagen (2.5 μg/mL)-induced platelet aggregation. Even at 1 mg/mL, the peptide showed only 10.46 ± 4.12% and 2.44 ± 1.65% inhibitory rates (mean ± SD, n = 3) against thrombin (3 U/mL)- and collagen (2.5 μg/mL)-induced rabbit platelet aggregation, respectively.

### ACH-11 inhibited ADP-induced acute pulmonary thrombosis in mice and arterio-venous shunt thrombosis in rats *in vivo*

To examine whether ACH-11 exerted antithrombotic effects *in vivo*, the peptide was challenged in an acute pulmonary thrombosis model in mice. Aspirin, which is a clinical anti-thrombosis medicine that acts as a selective COX-1 inhibitor, was used as a positive control. In this model, small thrombi formed by intravenous injection of ADP (300 mg/kg) occluded pulmonary vessels, resulting in paralysis or death of the mice. These results showed that intravenous pre-administration of the peptide protected against lethality in a dose-dependent manner (Table 1). Administration of the peptide at 3 mg/kg (i.v.) significantly prevented paralysis and death, with a protection rate of 90%. Moreover, the peptide showed a more potent effect than aspirin, as aspirin showed only 80% protection rate with a 6-fold higher dose of 20 mg/kg.

**Figure 6:**
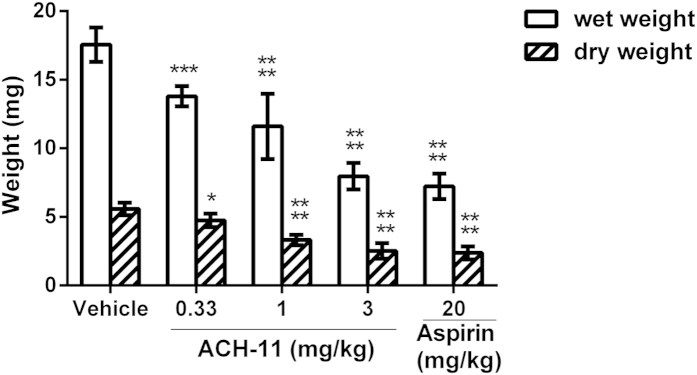
ACH-11 inhibited arterio-venous shunt thrombosis in rats *in vivo*. After the administration of ACH-11 (0.33, 1 and 3 mg/kg), aspirin and the vehicle, rats were anesthetized by intraperitoneal injection of chloral hydrate (350 mg/kg), and an arterio-venous shunt tube (12 cm, containing 10-cm long of 4 braided silk thread) was installed between the right carotid artery and left jugular vein of each rat. After 20 min, the silk thread was removed from the shunt tube, and its wet weight was immediately measured. The dry weight of the thread was measured 6 h later at room temperature. The wet and dry weights of the formed thrombi were determined by subtracting the pre-experiment weights of the wet and dry 6-cm threads, respectively. Data are the mean ± SD, ^*^P < 0.05, ^***^P < 0.001 and ^****^P < 0.0001 *versus* vehicle, n = 6, analyzed by one-way ANOVA, followed by the Tukey multiple comparison test.

The effect of ACH-11 on thrombus formation was further evaluated in the rat arterio-venous shunt thrombosis model. As illustrated in Fig. 6, ACH-11 inhibited thrombosis formation in a dose-dependent manner, while the anti-thrombosis ability of ACH-11 was better than that of aspirin. After administration of ACH-11 (i.v.) at 0.33, 1 and 3 mg/kg, thrombus weight was reduced by 14.93 ± 3.57%, 40.6 ± 2.75%, and 57.91 ± 2.80% (mean ± SEM, n = 6), respectively. However, when given 20 mg/kg of aspirin, the weight of the thrombus formed inside the arterio-venous shunt tube was reduced by 59.10 ± 2.98% (mean ± SEM, n = 6), indicating that a 6-fold higher dose of aspirin produced the same effect as ACH-11.

### ACH-11 exhibited a low bleeding risk in mice

**Figure 7:**
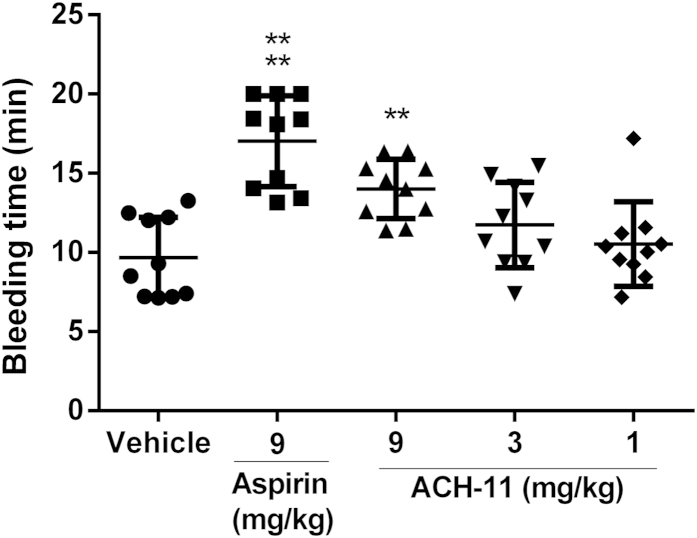
ACH-11 exhibited a low bleeding risk in mice. Fifteen minutes after the administration of ACH-11 (1, 3 and 9 mg/kg), aspirin (9 mg/kg for 30 min) and the vehicle, a 3 mm-long tail tip was cut from the mice, and the remaining tail was immersed immediately into saline at 37 °C. The accumulated bleeding time (including periods of re-bleeding) was recorded over a 20-min period. Data are presented as the mean ± SD (n = 10). ^****^P < 0.0001, ^**^P < 0.01 *versus* vehicle, analyzed by one-way ANOVA, followed by the Tukey multiple comparison test.

To assess the bleeding risk incurred by ACH-11, we measured the bleeding time of ACH-11-treated mice by a mice tail cutting assay at ACH-11 concentrations of 1, 3 and 9 mg/kg, representing three times the doses used for the *in vivo* anti-thrombotic studies. Aspirin (9 mg/kg)-treated mice served as the positive control. As shown in Fig. 7, a slight prolongation of the bleeding time was observed at 9 mg/kg of ACH-11 (14.01 ± 1.88 min, mean ± SD, n = 10), shorter than with aspirin (17.03 ± 2.86 min, mean ± SD, n = 10) treatment at the same dose. At doses of 3 and 1 mg/kg, the efficient dosages required to avoid thrombus formation and to protect against paralysis or death in mice, ACH-11 did not significantly prolong the bleeding time (11.73 ± 2.68 min and 10.71 ± 2.98 min, respectively, mean ± SD, n = 10) compared with the vehicle group, suggesting that ACH-11 confers a low bleeding risk.

## Discussion

Snake venoms have been a rich source of novel antithrombotic agents because they contain a variety of proteins and peptides that affect thrombosis and hemostasis^17^,^18^,^19^,^20^. The antithrombotic proteins and peptides from snake venoms have been extensively characterized over the previous several decades of research. Earlier studies have demonstrated that the bioactive peptides released from pro-proteins by enzymatic hydrolysis usually have multifunctional characteristics and are easily absorbed, so we aimed to discover small antithrombotic bioactive peptides by subjecting snake venom protein to enzymatic hydrolysis. The novel antithrombotic peptide named ACH-11 was isolated from *Agkistrodon acutus* venom hydrolysate, with the sequence LTFPRIVFVLG. ACH-11 exhibited both FXa inhibition and anti-platelet aggregation activities, and it showed potent antithrombotic activity *in vivo* in an acute pulmonary thrombosis model in mice and an arterio-venous shunt thrombosis model in rats. Interestingly, this peptide did not prolong bleeding times in mice at an effective antithrombotic dosage, indicating a favorable efficacy to bleeding ratio.

FXa is a key component in blood coagulation, and many FXa inhibitors have been isolated from animal venom, falling within a molecular mass range of 4–29 kDa, such as Ac-AP-12 (9.1 kDa) from the esophageal glands of adult *Ancylostoma caninum*^21^, Ruviprase (4423.6 Da) from the venom of *Daboia russelii russelii*^22^ and tick anticoagulant peptide (6 kDa) from the soft tick *Ornithodoros moubata*^23^. ACH-11, which consists of 11 amino acids with a MW of 1260.77 Da, is the smallest peptide identified from a natural source. Thus, it has substantial advantages over larger anticoagulant proteins, including low immunogenicity and low production costs.

The FXa inhibitors are classified into two groups: direct and indirect FXa inhibitors^7^. Direct FXa inhibitors are those that inhibit FXa directly, whereas indirect FXa inhibitors inhibit FXa activity with the aid of AT-III^24^. ACH-11 demonstrated direct inhibitory effects on FXa via a mixed model of inhibition, indicating that ACH-11 can inhibit free FXa or the complex of S-2222 and FXa, similar to the effect of Ruviprase, purified from Russell’s viper venom^22^, and AduNAP_4_, purified from the human hookworm^25^.

The anticoagulant activity of ACH-11 was verified using aPTT *ex vivo* assays and by examining clotting time of mice *in vivo*. To investigate whether the coagulation process was altered by ACH-11, we measured aPTT and PT to evaluate the intrinsic and extrinsic routes of coagulation, respectively. It was observed that treatment with ACH-11 induced an increase in aPTT as compared to the vehicle group, while no obvious increase in PT was found. The *in vivo* clotting time of ACH-11-treated mice was also prolonged in a dose-dependent manner.

ACH-11 was also a selective platelet aggregation inhibitor, as it inhibited ADP and U46619-induced rabbit platelet aggregation but did not inhibit collagen and thrombin-induced platelet aggregation. A large amount of clinical data^26^ have shown that the combination of antiaggregatory (e.g., acetylsalicylic acid or ticlopidine) and anticoagulant agents (e.g., warfarin or heparin), as well as combinations of thrombin or FXa inhibitors with GPIIb/IIIa antagonists, has an additive effect by suppressing both blood coagulation and platelet aggregation. Thus, this treatment is more effective than treatment directed against coagulation system or platelets alone^27^,^28^. ACH-11, a natural bioactive peptide possessing anti-FXa and antiaggregatory activities in a single molecule, may have substantial advantages over combination therapies, including greater efficacy *in vivo* and more predictable and less complex pharmacokinetics and pharmacodynamics.

*In vivo* antithrombotic effects of ACH-11 were observed in an acute pulmonary thrombosis model in mice and an arterio-venous shunt thrombosis model in rats. Pulmonary thrombosis is a common model in which platelet activator is injected into mice to cause formation of thrombi. This model is characterized by the massive activation of circulating platelets and the widespread formation of platelet thrombi in microcirculation in the lungs, leading to disseminated pulmonary microembolism and paralysis or death of the mice^29^. The arterio-venous shunt model produces a mixed thrombus of platelets and fibrin, in which both the coagulation cascade and the activation of platelets are determinants of thrombus formation. Developing thrombi in this model contain large platelet aggregation that adheres to the thrombogenic silk thread, surrounded by erythrocytes and fibrin^30^,^31^. ACH-11 exhibited a large antithrombotic effect *in vivo* in these two models, more so than in the anti-platelet aggregation assay *in vitro*. Provided that the average blood volumes of a 200 g rat and 20 g mouse were 15 mL and 2 mL, respectively, ACH-11 administered at 3 mg/kg produced a concentration of approximately 0.04 and 0.03 mg/mL in rat and mouse peripheral blood, respectively. Compared with *in vitro* data, it is noteworthy that ACH-11 inhibition of platelet activity *in vivo* was more potent than when incubated with rabbit PRP, in which ACH-11 at a concentration of 0.1 mg/mL inhibited ADP-induced platelet aggregation by 32.82%. The effective concentration affecting anti-platelet aggregation *in vitro* was higher than that in systemic circulation in the animal model, further confirming that ACH-11 not only inhibited platelets but also affected other pathways of thrombosis.

Thrombosis-associated disorders such as cardiovascular disease are a leading cause of mortality throughout the world^32^. Unfortunately, many antithrombotic drugs are associated with side effects, which primarily manifest as bleeding complications. At a dose of 3 mg/kg, ACH-11 demonstrated strong antithrombotic activity without a significant bleeding risk. Similar results were observed after combined therapy with direct FXa inhibitors and anti-platelet agents^33^,^34^. For example, the combination of apixaban and aspirin reduced the formation of occlusive arterial thrombosis with only a small increase in bleeding time^35^. We also tested the thrombin inhibitory activity of ACH-11, and our results showed that it had no inhibitory effect on the amidolytic activity of thrombin (data not shown). As ACH-11 selectively inhibits FXa amidolytic activity without affecting the function of thrombin in the circulation system, it should have a minimal impact on normal hemostatic response and regulation processes. The activation of the GPIIb/IIIa receptor is the common pathway for platelet aggregation, and many of its antagonists exhibit a severe bleeding risk. A GPIIb/IIIa-fibrinogen binding assay showed that ACH-11 was not an antagonist of GPIIb/IIIa (data not shown).

In conclusion, ACH-11 is a novel antithrombotic peptide isolated from *Agkistrodon acutus* venom hydrolysate. ACH-11 combines FXa inhibitory and anti-platelet activities into a single molecule, showing potent antithrombotic activity *in vivo* without an obvious bleeding risk. The mechanism of ACH-11 inhibition of platelet aggregation and its pharmacokinetics and pharmacodynamics *in vivo* are under investigation.

## Methods

### Preparation of *Agkistrodon acutus* venom hydrolysates

*Agkistrodon acutus* venom (0.4 g) was dissolved in 10 mL of 0.1 M phosphate buffer solution (PBS) (when using neutrase, papain and alcalase) or 0.1 M Glycine-HCl solution (when using pepsin). Then, it was hydrolyzed for 12 h using neutrase at pH 8.0 at 50 °C, alcalase at pH 8.0, 50 °C, pepsin at pH 2.0, 37 °C and papain at pH 6.0, 37 °C with a total enzyme dose of 1%^15^. Aliquots of each hydrolysate were collected at 1 h, 2 h, 3 h, 4 h, 8 h and 12 h and were boiled for 10 min to stop the reaction. The final hydrolysates were centrifuged at 10,000 rpm for 10 min, and the supernatants were freeze-dried.

### Anticoagulant peptide purification

The lyophilized neutrase hydrolysate (0.2 g) was dissolved in 10 mL of distilled water and applied to a Sephadex G-50 column (2.6 × 100 cm) equilibrated with distilled water and eluted with the same buffer at a flow rate of 0.6 mL/min. The eluents were monitored at 280 nm and were collected at 6 min intervals. The fractions were tested for FXa inhibitory activity. The fraction that showed the strongest FXa inhibitory activity was further separated by RP-HPLC on a Lichrospher C_18_ column (20 × 250 mm; Hedera, China) under linear gradient elution conditions using acetonitrile as the organic modifier and trifluoroacetic acid (TFA) as the volatile buffer. Eluent A was consisted of 0.1% TFA in 10% acetonitrile (v/v), and eluent B consisted of 0.1% TFA in 90% acetonitrile (v/v). Gradient elution was carried out according to the following process: 0–15 min, B 10%, and 10–60 min, B 10–30%. The flow rate was 7 mL/min. The UV absorbance was monitored at 214 nm. The fractions were tested for FXa inhibitory activity, and the fraction that showed the strongest FXa inhibitory activity (fraction C4) was pooled and lyophilized.

### Inhibition of amidolytic activity of FXa

Inhibition of the amidolytic activity of FXa was analyzed by a method described previously, with minor modifications^36^. Enzymatic activity was measured in PBS at pH 8.34 using the chromogenic substrate method. Freeze-dried samples were dissolved in distilled water at a concentration of 5 mg/mL. The sample in 10 μL was added to 50 μL of 1 nM enzyme solution (FXa) and preincubated at 37 °C for 30 min. The reaction was initiated by the addition of 40 μL of 300 μM S-2222 substrate solution that was preincubated at 37 °C, and the color was monitored continuously at 405 nm using Powerwave XS (Biotek, USA) for 20 min. The absorbance-time curve and the slope of the curve (V_i_) were used to represent the activity of the enzyme. Distilled water served as a control (the slope of curve V_0_). The inhibitory effect was calculated according to equation (1):
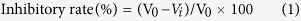
where V_0_ represents the slope of the vehicle and V_i_ represents the slope of the samples.

### Determination of molecular mass and peptide sequence

The molecular mass and amino acid sequence of the purified peptide were determined by electrospray ionization-tandem mass spectrometry (ESI-MS/MS) on an Agilent 6520 accurate-mass Q-TOF mass spectrometer (Agilent, USA) equipped with an electrospray ion source in positive ion mode. The freeze-dried sample obtained by RP-HPLC was dissolved in distilled water containing 5% DMSO. The solution was injected continuously, and the setting parameters were 40 psi ESI nebulizer gas (N_2_) pressure, 8.00 L/min ESI drying gas flow rate operating on positive mode with a capillary voltage of 4000 V and capillary temperature of 350 °C, and a fragmentor voltage of 120 V. The MS/MS spectra were acquired in the data-dependent mode, in which collision energy was 70 V. Positive ion intensities were recorded over a mass range (m/z) of 100–1700. A *de novo* peptide sequencing method was performed by a manual calculation.

### Peptide synthesis

Solid-phase peptide synthesis was performed according to the method described previously^37^ (supplementary information).

### Animals

Institute of Cancer Research (ICR) mice (18–22 g), Sprague-Dawley rats (180–250 g) and New Zealand white rabbits (2–2.5 kg) were purchased from Nanjing Qinglongshan Animal Center (Jiangsu province, China). They were housed in a temperature-controlled room at 25 ± 2 °C and 50 ± 5% relative humidity with a l2 h dark/light cycle. They were given free access to food and water *ad libitum*. The animals were acclimatized for at least 1 week prior to use. All experiments were carried out in accordance with the guidelines and the regulations of the Ethical Committee of the China Pharmaceutical University. The protocols were approved by the Institutional Animal Care and Use Committee of the China Pharmaceutical University.

### *In vitro* platelet aggregation assay

*In vitro* platelet aggregation assays were performed according to the turbidimetric method, as described previously^37^. Sample solutions of ACH-11 were tested at concentrations of 200, 400, 600, 800 and 1,000 μg/mL. DMSO (5%) served as the vehicle. The extent of platelet aggregation inhibition by ACH-11 was calculated by the following formula (2):
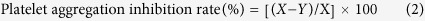
where X represents the maximum aggregation rate of 5% DMSO-treated PRP and Y represents the maximum aggregation rate of sample-treated PRP. IC_50_ was defined as the drug concentration required to inhibit platelet aggregation by 50%.

### Determination of the inhibitory constant for FXa

Enzyme kinetic activity was assayed as described earlier, with minor modifications^38^. All enzymatic reactions were carried out in PBS at pH 8.34 and at 37 °C. Freshly prepared FXa at a constant concentration of 1 nM was used in each reaction. ACH-11 was dissolved in 5% DMSO. Activities were measured for 4 different fixed concentrations of substrate (73.5, 147, 294 and 588 μM) in different concentrations of ACH-11 (0, 5 and 10 μM). The rates of the enzymatic reactions were monitored at 405 nm for 20 min using the Powerwave XS (Biotek, USA).

### ADP-induced acute pulmonary thrombosis in mice

ADP-induced acute pulmonary thrombosis in mice was performed as described previously^37^. Sixty ICR mice (18–22 g) were fasted overnight and randomly divided into 6 groups, with 10 mice per group (males and females in half). The mice were intravenously injected with ACH-11 at doses of 0.33, 1 and 3 mg/kg, aspirin (20 mg/kg) or with the vehicle.

### *In vivo* arterio-venous shunt thrombosis

Antithrombotic activity of ACH-11 *in vivo* was determined in a rat arterio-venous shunt thrombosis model as described previously^39^ with a minor modification. Sprague-Dawley rats were weighed and randomized into 5 groups (6 rats per group), including a vehicle group, a reference drug group (20 mg/kg of aspirin-treated), and three sample groups (0.33, 1 and 3 mg/kg of ACH-11-treated, 5 mL/kg). Rats were anesthetized by chloral hydrate (350 mg/kg, i.p.), and an arterio-venous shunt tube was installed between the right carotid artery and the left jugular vein^40^,^41^. The polyethylene tube (12 cm; containing 10-cm long of 4 braided silk thread) was filled with a heparin saline solution (25 IU/mL) prior to installation. After blood circulation through the shunt tube for 20 minutes, both ends of the tube were pinched, and the silk thread was removed from the shunt tube. Its wet weight was measured immediately. The dry weight of thread was measured 6 h later at room temperature. The wet and dry weights of the thrombi were determined by subtracting the pre-experimental weights of the wet and dry 6-cm threads, respectively.

### *Ex vivo* coagulation parameters

Rat grouping, dosage and administration in this assay were the same as in the arterio-venous shunt thrombosis experiments. Fifteen minutes after the administration of ACH-11 (0.33, 1 and 3 mg/kg) or saline, blood was take n from the inferior vena cava, and plasma was then obtained by centrifuging at 2,000 g for 10 min at room temperature in order to measure PT and aPTT. The coagulation parameters were determined by routine laboratory assays using an automated coagulation analysis instrument (Coatron M4, TECO GmbH, Germany). For the measurement of aPTT, 50 μL of plasma was preincubated for 5 min at 37 °C, and 50 μL of aPTT reagent was then added and incubated for another 5 min, followed by the addition of 50 μL of 25 mM CaCl_2_ solution. To measure PT, 50 μL of plasma was preincubated for 5 min at 37 °C, which was followed by the addition of 100 μL prothrombin reagent. The coagulometer was started, and time to clot formation was recorded.

### Clotting time *in vivo*

Whole blood clotting time (CT) in mice was measured by a capillary glass tube method as described previously^42^. Mice were randomly divided into four groups (both sexes, 10 per group). Three groups were i.v. injected with 0.33, 1 and 3 mg/kg body weight of ACH-11 for four consecutive days. The other group received the same volumes of the vehicle.

### Bleeding time

Bleeding time of the mice was measured by a tail cutting method as described previously^42^. Mice were randomly divided into five groups (both sexes, 10 per group), including a vehicle group, a reference drug group (9 mg/kg of aspirin-treated) and three sample groups (1, 3 and 9 mg/kg of ACH-11-treated).

### Statistical analysis

The data are expressed as the mean of the replicate determinations and the standard deviation (SD) or standard error of measurement (SEM). Statistical significance was evaluated using ANOVA, followed by Tukey’s test for multiple comparisons. Differences between groups were analyzed by Fisher’s test for pulmonary thrombosis. The data were analyzed using GraphPad Prism 5 (GraphPad Software, Inc., La Jolla, CA, USA).

## Additional Information

**How to cite this article**: Chen, M. *et al.* A Novel Direct Factor Xa Inhibitory Peptide with Anti-Platelet Aggregation Activity from *Agkistrodon acutus* Venom Hydrolysates. *Sci. Rep.* **5**, 10846; doi: 10.1038/srep10846 (2015).

## Supplementary Material

Supplementary Information

## Figures and Tables

**Table 1:** ACH-11 inhibited ADP-induced acute pulmonary thrombosis in mice.

**Samples**	**Dose for mice (mg/kg)**	**Numbers of lethal/total**	**Protection rate (%)**	**P values (compared with vehicle)**
Vehicle		9/10	10	
Aspirin	20	2/10	80^**^	0.0055
ACH-11	0.33	7/10	30	0.5820
	1	4/10	60^*^	0.0198
	3	1/10	90^**^	0.0011

Note: ^**^P < 0.01, ^*^P < 0.05 *versus* vehicle, n = 10 (χ^2^ test).
