# Supplementary material for: A Novel Direct Factor Xa Inhibitory Peptide with Anti-Platelet Aggregation Activity from Agkistrodon acutus Venom Hydrolysates
Source: Sci Rep. 2015 Jun 2;5:10846. doi: 10.1038/srep10846 (PMC4451689; doi:10.1038/srep10846)
Supplement: Supplementary Information [file srep10846-s1.pdf]

A Novel Direct Factor Xa Inhibitory Peptide with Anti-Platelet Aggregation Activity from *Agkistrodon acutus* Venom Hydrolysates

Meimei Chen[1], Xiaohui Ye[1], Xin Ming[3], Yahui Chen[1], Ying Wang[1], Xingli Su[1], Wen Su[1] & Yi Kong[1,2*]

[1]School of Life Science & Technology, China Pharmaceutical University, 24 Tong Jia Street, Nanjing 210009, PR China, [2]State Key Laboratory of Natural Medicines, China Pharmaceutical University, Nanjing 210009, PR China, [3]Division of Molecular Pharmaceutics, UNC Eshelman School of Pharmacy, The University of North Carolina at Chapel Hill, Chapel Hill, NC 27599, USA.

*** Corresponding to**

Dr. Yi Kong, School of Life Science & Technology, China Pharmaceutical University, 24 Tong Jia Street, Nanjing 210009, PR China.

Tel: 0086-25-83271282, Fax: 0086-25-83271282. E-mail: yikong668@163.com.

SUPPLEMENTARY INFORMATION

Peptide synthesis

The peptide used for the bioactivity assays was synthesized using the Fmoc solid-phase synthesis method. The peptide was synthesized on the 2-chlorotrityl chloride resin, and the Fmoc-protected amino acids were successively coupled in the presence of HBTU and HOBT. The washing reagent was DMF and the deprotection reagent was 20% piperidine in DMF. The peptide was cleaved by a mixture of TFA/Tis/water/EDT (94/1/2.5/2.5, v/v/v/v) at 4°C for 1.5 h. After being cut off from the resin, the synthesized peptide was purified by the BioLogic Duoflow system (Bio-Rad, USA) on a Kromasil Semipreparative C_{18} column (10 × 250 mm, Hanbon, China). The peptide was validated by ESI-MS, then it was concentrated, lyophilized and stored at -20°C.

Supplementary Figure: S1

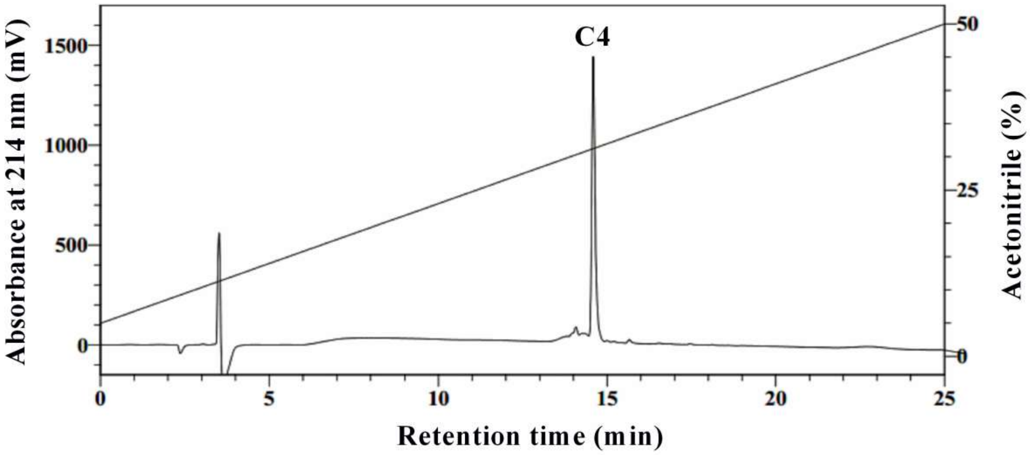

The purity of the fraction C4 measured by analytical RP-HPLC. It was performed on a Lichrosper C_8 column (4.6 × 250 mm) with a linear gradient elution conditions using acetonitrile as the organic modifier and trifluoroacetic acid (TFA) as the volatile buffer. Eluent A was consisted of 0.1% TFA in 10% acetonitrile (v/v), and eluent B of 0.1% TFA in 90% acetonitrile (v/v). Gradient elution was carried out according to the following process: 0-25 min, B 5-50%. A single prominent

peak was observed at retention time of 14.62 min. The purity was above 96%.

Supplementary Figure: S2

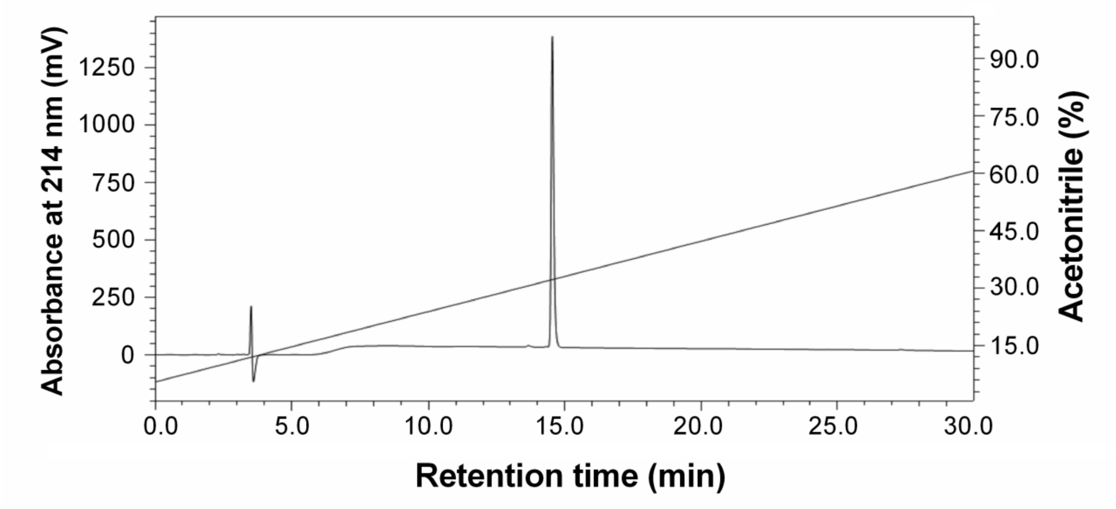

The purity of ACH-11 synthesized using the Fmoc solid-phase synthesis method was measured by analytical RP-HPLC. It was performed on a Lichrosper C_8 column (4.6 × 250 mm) with a linear gradient elution conditions using acetonitrile as the organic modifier and trifluoroacetic acid (TFA) as the volatile buffer. Eluent A was consisted of 0.1% TFA in 10% acetonitrile (v/v), and eluent B of 0.1% TFA in 90% acetonitrile (v/v). Gradient elution was carried out according to the following process: 0-30 min, B 10-60%. A single prominent peak was observed at retention time of 14.68 min. The purity was above 98%.

Supplementary Figure: S3

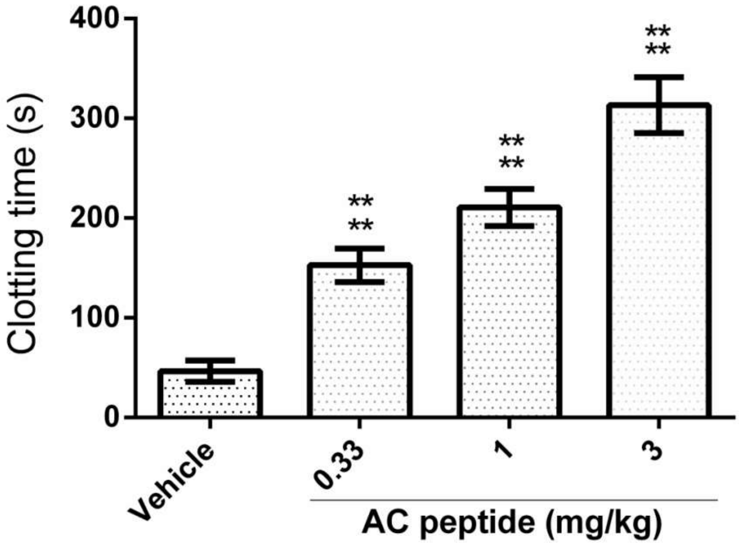

ACH-11 prolonged clotting time of mice *in vivo*. It was measured by capillary glass tube method. Mice (18-22 g body weight) were randomly divided into four groups (both sexes, 10 per group). Three groups were received intravenous injection of 0.33, 1 and 3 mg/kg body weight of ACH-11 for four consecutive days. The other group was received the same volumes of vehicle. Fifteen minutes after the last administration, blood samples were collected through the retro-orbital plexus with a glass capillary and kept on a slide to allow for clotting. Stirring the blood with a dry needle every 30 s until needle wire can provoke a fibrous protein, when is defined as clotting time. ****P < 0.0001 *versus* vehicle, n=10, analyzed by one-way ANOVA, followed by the Tukey multiple comparison test.
